# Long term monitoring and adaptive strategies: lessons from Hainan’s malaria elimination and prevention efforts

**DOI:** 10.1136/bmj-2024-080657

**Published:** 2025-04-22

**Authors:** Ziyan Liu, Oliver J Brady, Guangze Wang, Yapin Li, Nils Chr Stenseth, Christopher Dye, Qiyong Liu, Huaiyu Tian

**Affiliations:** 1 Beijing Key Laboratory of Surveillance, Early Warning and Pathogen Research on Emerging Infectious Diseases, Beijing Research Center for Respiratory Infectious Diseases, National Key Laboratory of Intelligent Tracking and Forecasting for Infectious Diseases, Center for Global Change and Public Health, Beijing Normal University, Beijing, China; 2 Centre for the Mathematical Modelling of Infectious Diseases, London School of Hygiene and Tropical Medicine, London, UK; 3 Department of Infectious Disease Epidemiology and Dynamics, Faculty of Epidemiology and Public Health, London School of Hygiene and Tropical Medicine, London, UK; 4 Hainan Center for Disease Control and Prevention, Hainan Academy of Preventive Medicine, Haikou, China; 5 Central Theater Center for Disease Control and Prevention of PLA, Beijing, China; 6 The Centre for Pandemics and One-Health Research, Sustainable Health Unit, Faculty of Medicine, University of Oslo, Oslo, Norway; 7 Centre for Ecological and Evolutionary Synthesis, Department of Biosciences, Faculty of Mathematics and Natural Sciences, University of Oslo, Oslo, Norway; 8 Vanke School of Public Health, Tsinghua University, Beijing, China; 9 Department of Biology, University of Oxford, Oxford, UK; 10 National Key Laboratory of Intelligent Tracking and Forecasting for Infectious Diseases, National Institute for Communicable Disease Control and Prevention, Chinese Center for Disease Control and Prevention, WHO Collaborating Centre for Vector Surveillance and Management, Beijing, China

## Abstract

**Huaiyu Tian and colleagues** argue that to improve sustainable malaria control and reduce the risk of disease resurgence, targeted interventions can be optimised for eliminating malaria in areas co-endemic for multiple Plasmodium species

The intensity of malaria transmission is influenced by factors related to the parasite, the human host, and the *Anopheles* mosquito and which of these factors become targets of intervention strategies that aim to treat and prevent infection and disease.^1^ ^2^ Although the World Health Organization has certified 40 countries and territories, including China, as malaria free in 2021,^3^ evidence to guide elimination planning is lacking in more than half of the cases. WHO defines malaria elimination as the interruption of local transmission of a specified malaria parasite species in a defined geographic area through deliberate activities.^4^ Malaria prevalence and interventions have rarely been meticulously documented throughout the long elimination process, leading to a lack of evidence to guide elimination planning.

We aimed to analyse the malaria elimination programme in Hainan, China, between 1959 and 2021 to highlight two topics of relevance to today’s global malaria strategy: how to target malaria elimination in high burden areas where the disease remains endemic and how to prevent re-emergence of malaria in areas where it has been eliminated.^5^ Selecting the optimal combination of interventions and prioritising these to maximise impact are critical to current elimination efforts.

## Hainan: among the provinces most severely affected by malaria in China

Hainan, an island province located in southern China, consists of Hainan Island and several smaller surrounding islands. The province has a warm, tropical climate, characterised by substantial precipitation, with annual rainfall ranging from 1000 to 2600 mm and an average annual temperature of 22-27°C. These climatic conditions create an ideal environment for *Anopheles* mosquitoes. Entomological surveys have shown that the abundance of the mosquito population has remained relatively constant over time.^6^ ^7^ The baseline infection rates of *Anopheles* mosquitoes in Hainan were significantly higher than in regions with similar malaria transmission environments such as Myanmar.^8^ Hainan’s climate and environment create favourable conditions for year round malaria transmission, making malaria elimination unlikely to succeed.

**Fig 1:**
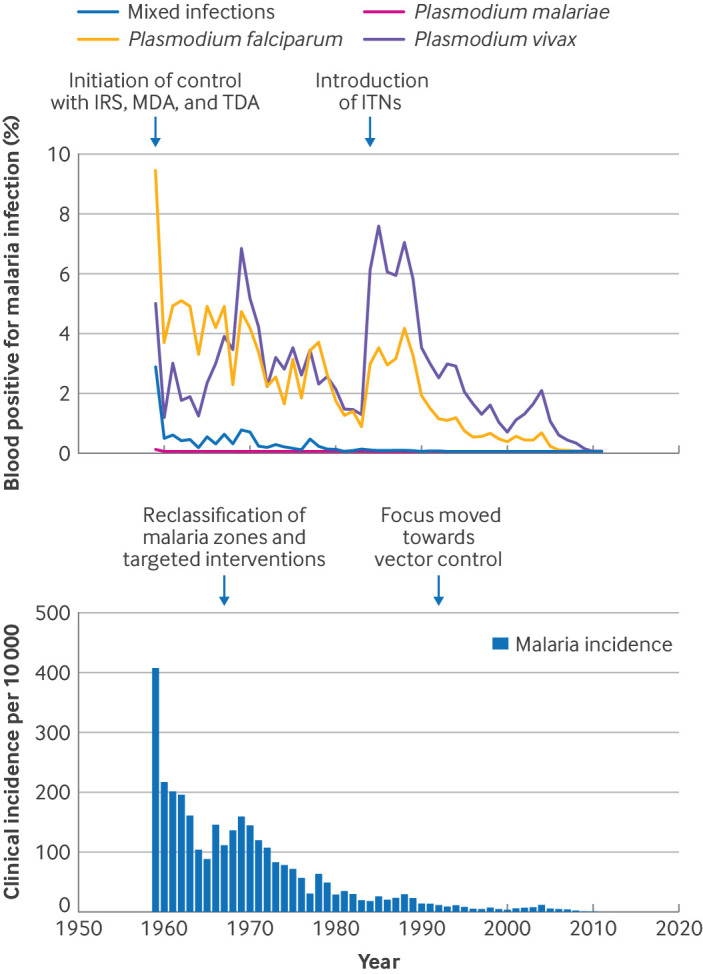
Malaria epidemics in Hainan between 1959 and elimination of endemic malaria in 2011. Top: lines correspond to changes in positive rates of *Plasmodium vivax*, *P malariae*, *P falciparum*, and mixed infection in sample of general population measured via annual blood examinations. Thick lines show blood positivity for malaria infection. Bottom: bars represent annual malaria incidence. Vertical dashed lines indicate timing of start of four main intervention strategies successfully introduced: indoor residual spraying (IRS), mass drug administration (MDA), targeted drug administration (TDA), and insecticide treated bed nets (ITNs), which were gradually introduced and became widely used in Hainan as malaria control measures

From 1959 to 2011, more than 1.3 million cases of clinical malaria were reported in Hainan, with an average annual incidence rate of 5.91 cases per million population (fig 1). Over the past 53 years, the province has reduced the prevalence of malaria from 30% to zero,^4^ demonstrating the effectiveness of its tailored strategies. Building on the elimination of indigenous transmission in 2011, strategies have since focused on strengthening the malaria prevention infrastructure, prioritising the mitigation of risks of reintroduction, and maintaining zero transmission. This achievement aligns with WHO’s goal of eliminating malaria in at least 30 countries by 2030 and underscores the importance of adapting interventions to specific local conditions.^9^ The strategic flexibility in Hainan—continually refining intervention methods as transmission decreased—has been a key factor in its success and offers a model for other highly endemic regions.

## Adaptive intervention strategies for malaria elimination in Hainan

Hainan’s malaria elimination efforts have been characterised by adaptive strategies tailored to a changing endemic situation (table 1). Central to these efforts has been timely epidemiological investigation of malaria cases and foci conducted by the Centers for Disease Control (CDCs) in various cities and counties. In the early morbidity and infection control stages, when the disease burden is high, mass drug administration and indoor residual spraying were prioritised to rapidly reduce transmission. As the malaria burden declined, interventions shifted during the transmission control stage to more targeted approaches, such as targeted drug administration and the distribution of insecticide treated bed nets. Finally, the CDC implemented the “1-3-7” malaria case management approach in the transmission interruption stage, which mandates case reporting within one day, investigation within three days, and a targeted response within seven days (see other articles in this collection for more information). These adaptive strategies aimed to follow the principles of the WHO Global Technical Strategy for Malaria,^4^ combining targeted interventions with innovative tools and implementation methods.

**Table 1:** Key elimination interventions in Hainan

Intervention strategies	Mechanism of action	Implementation period	Target population/area	Materials/insecticide used	Coverage/effectiveness	Supplementary notes
Indoor residual spraying	Kills adult mosquitoes resting on walls before or shortly after blood meal	Spring and autumn	Ultra-high and high malaria areas	6% deltamethrin (240 mg/m²), or 6% lambda-cyhalothrin (240 mg/m²), or 25% DDT (2 g/m)	All dwellings in targeted areas	Local government selects insecticide on basis of vector species and resistance monitoring
Insecticide treated bed nets	Prevents adult mosquitoes from delivering infectious bites as long as person is under net; kills adult mosquitoes, offering individual and community protection	Annual peak malaria season (usually in April)	Malaria endemic areas; residential sites	30 mg permethrin or 15 mg cyfluthrin/m^2^ of net	Coverage protection rate: >70% in residential areas; >90% for impregnated/sprayed nets	Supplemented with indoor residual spraying in areas with coverage <70%
Mass drug administration	Reduces parasite population through monthly treatment	April to November	Entire population (>90% coverage)	Two day intermittent treatment with 50 mg piperaquine and 22.5 mg primaquine; short term and intensive therapy that rapidly reduces number of parasites in patient’s body	Achieves elimination through pharmacological action with continuous dosing	Combination of antimalarial drugs can be adjusted to piperaquine (150 mg) and primaquine (22.5 mg) on basis of sensitivity monitoring
Targeted drug administration	Prevents transmission among high risk groups; treats existing infections	Spring (by May) and autumn (by September)	Individuals/groups at high risk (eg, recovered malaria cases, people whose occupation puts them at increased risk of malaria infection)	Piperaquine (1-10 tablets) combined with primaquine (24 tablets for four day course and 12 tablets for eight day course)	Drug uptake should be ≥90%	Administered before, during, or after exposure to malaria transmission

DDT=dichlorodiphenyltrichloroethane.

Initially, malaria interventions in Hainan were not rigidly tied to a single stage of the elimination process but were adapted to respond to immediate needs at various points in time. Since 1953 the Hainan Malaria Research Station of the Chinese Academy of Medical Sciences has conducted detailed surveys to map malaria prevalence, informing strategic decisions. In 1958 a comprehensive malaria control campaign was initiated by the Hainan government, with Lingshui and Baoting counties serving as pilot sites. By 1967 Hainan authorities had reclassified malaria zones on the basis of risk of transmission, enabling the implementation of targeted interventions such as indoor residual spraying with DDT (dichlorodiphenyltrichloroethane) and the deployment of piperaquine-primaquine combinations for mass drug administration and targeted drug administration. In 1992 the focus of malaria management moved towards vector control, primarily through the widespread use of deltamethrin treated bed nets. From 2003 to 2011 Hainan further advanced its comprehensive malaria elimination strategy, incorporating seasonal campaigns that targeted high risk groups during peak transmission periods in spring and autumn. After 2011 Hainan authorities focused on maintaining elimination by strengthening surveillance, rapidly responding to imported cases, and maintaining public awareness campaigns.

## Key strategies for successful malaria elimination in Hainan

### Apply a comprehensive system of vector control and antimalarial drug use

Hainan’s success in eliminating malaria can be attributed to the sustained effectiveness of a comprehensive long term approach. Malaria elimination programmes have been regularly carried out in Hainan since 1959, with priority given to indoor residual spraying and mass drug administration. However, the implementation of malaria interventions in Hainan has faced enormous challenges, including inadequate antimalarial resources, insufficient adaptation to climate change, incomplete coverage of preventive measures among the population, and drug resistance. For example, owing to funding adjustments and a shortage of technical personnel, the residual *An minimus* capture rate rebounded to 28.25% in 1978, significantly higher than the 0.91% rate in 1964, leading to ineffective malaria control.^10^ In 1994 an early onset of the malaria transmission season owing to the warming climate led to a 19.18% increase in the malaria incidence compared with 1993.^10^ Additionally, in 2000 the malaria mortality rate rose to 0.08 per million, driven by the influx of non-immune migrant workers from other provinces into the *P falciparum* endemic regions of central and southern Hainan.^11^ Malaria control was subsequently improved through better targeting of elimination strategies supplemented by the joint use of insecticide treated bed nets and targeted drug administration. The case of Comoros, Africa, highlights the importance of surveillance for maintaining suppression of transmission. Although mass drug administration in Comoros initially achieved an impressive 95% reduction in malaria cases, the absence of sustained surveillance and complementary interventions led to a resurgence of cases.^12^ By contrast, Hainan’s success stems from combining mass drug administration and indoor residual spraying with consistent surveillance and targeted interventions,^13^ ^14^ ^15^ effectively preventing resurgence of transmission.

### Establish feasible mosquito vector control programmes

The promotion and widespread use of insecticide treated bed nets have long been a cornerstone of effective vector control, leading to significant reductions in the malaria incidence in regions where they were implemented. Notably, China, including Hainan, was the first country to extensively adopt widespread use of insecticide treated bed nets as a malaria control measure before it was adopted in sub-Saharan Africa.^16^ However, recognising the constraints of limited resources, Hainan adapted its approach by shifting to a coverage ratio of one net per three individuals, slightly below the WHO recommended rate (one net per 1.8 individuals).^17^ This adjustment proved to be both economically viable and highly effective in the local context, particularly in reducing costs while maintaining sufficient protection. By 2011 Hainan’s insecticide treated bed net coverage rate was reported to be one net per 1.97 (range 1.05-2.78) individuals. This more flexible approach allowed for broader distribution of insecticide treated bed nets without compromising the core goal of reducing malaria transmission. The success of this model is evidenced by the significant reduction in malaria incidence, with the introduction of deltamethrin treated nets in the 1990s, resulting in a 77.5% decrease in cases within five years.^10^

Furthermore, high coverage of insecticide treated bed nets in malaria endemic areas can provide protection for people who do not use them directly by decreasing mosquito populations.^18^ ^19^ ^20^ ^21^ Despite huge efforts to deploy insecticide treated bed nets across sub-Saharan Africa, the coverage remains uneven. By 2019 only 36% of households had access to at least one insecticide treated bed net per two individuals.^22^ Prompt adjustment, such as modifying the recommended coverage ratio to align with local resource availability, increases the protective benefits of mosquito vector control for users of insecticide treated bed nets and ensures broader distribution within budgetary constraints. The example of Hainan emphasises the importance of local adaptation of global recommendations and shows how, despite resource constraints, significant public health benefits can be made while gradually building up to more ambitious coverage targets.

### Engage continuous drug management strategies

Achieving malaria elimination in the context of escalating drug resistance requires continuous and region specific drug administration strategies, as demonstrated in Hainan. Dispersed populations and limited healthcare infrastructure complicate the implementation and monitoring of interventions in malaria-prone areas, such as mountainous and hilly regions. Healthcare facilities at all levels throughout Hainan conduct regular rapid malaria diagnostics in high risk areas, monitor the type of *Plasmodium* infection, and administer drugs to specific populations. The need to comprehensively monitor and tackle safety, efficacy, and resistance concerns associated with new malaria treatments was emphasised by the Mpumalanga Province trial in South Africa.^23^ This trial specifically focused on emerging drug resistance patterns and assessed the real world safety and efficacy of antimalarial drugs.

Healthcare facilities at all levels have adopted an approach of using combination or complementary drugs to deal with the problem of drug resistance associated with continuous use of a single drug. This approach involves periodically changing or supplementing drug combinations, with resistance gradually declining after re-testing, before returning to the original regimen. Since the first case of chloroquine resistant *P falciparum* was detected in Sanya, Hainan, in 1974, the prevalence of chloroquine resistant *P falciparum* in Hainan has exceeded 60%. Regular monitoring in Ledong County since 1981 has shown a slow decline in resistance rates following the discontinuation of chloroquine in 1979, with resistance dropping from 97.3% in the early 1980s to 53.7% in the mid-1990s,^11^ indicating a gradual recovery of susceptibility. However, resistance to piperaquine has increased owing to its widespread use. Implementing a strict drug regimen and prohibiting the misuse of antimalarials have been effective in slowing the development of resistance. For instance, artemisinin resistant *P falciparum* has been detected at the Cambodia-Thailand and Thailand-Myanmar borders, although no resistance has been observed at the China-Myanmar border. This may be a result of China’s stricter malaria control measures, including enhanced monitoring and drug management policies.^24^

### Sustain malaria control with funding from multiple sources

Hainan’s success in malaria elimination has been supported by robust and diverse funding and socioeconomic improvements. In 1984 Hainan expanded its malaria control efforts with a five year grant from WHO’s Special Programme for Research and Training in Tropical Diseases. Since April 2003 the province has benefitted from the Global Fund Malaria Programme, including participation in the first and fifth rounds of the National Malaria Elimination Strategy Programme. Sustained funding from multiple sources has been crucial to reducing malaria transmission, developing the health workforce, and maintaining these gains over time (see other articles in this collection). Furthermore, reduced poverty, improved living standards, and enhanced healthcare have played a supportive role in sustaining malaria control efforts.

## Keys to successful resurgence prevention in Hainan

### Maintain a high level of surveillance and emergency response capacity

Despite the successful elimination of malaria in Hainan, the presence of residual malaria vectors and occasional imported cases underscore the continuing risk of malaria resurgence. Having achieved zero clinical malaria infections, the Hainan Health Commission has intensified surveillance of imported cases and rapid emergency responses, focusing on early detection, treatment, prevention, and the rapid deployment of targeted drug administration in the event of a possible concentrated malaria outbreak. By redefining malaria endemic areas and targeting the characteristics of the incidence of malaria in different regions, as well as the current status of malaria prevention, control, and surveillance efforts, the strategy has shifted from “reducing incidence in high risk areas” to “controlling each case and outbreak site to interrupt malaria transmission.” This approach is based on the principles of tailoring interventions to local conditions and providing categorised guidance; it builds on a successful pilot project in Tanzania between 2015 and 2018, in which China’s 1-3-7 approach was adapted to high transmission areas to establish Tanzania’s 1.7 Malaria Reactive Community Testing and Response method.^25^

### Strengthen control of *Anopheles* mosquito vector, even after elimination of malaria

An important challenge in sustaining malaria elimination in Hainan lies in the absence of consistently effective insecticides to prevent the disease. As a result, the cornerstone of preventing malaria resurgence has relied heavily on robust vector control measures. These measures have included the widespread use of insecticide treated bed nets, indoor residual spraying, and continuous monitoring of mosquito populations to adapt strategies in response to changes in vector behaviour or resistance patterns. When local or imported malaria cases are detected, the local CDC conducts indoor residual spraying with insecticides such as deltamethrin, malathion, or DDT (government approval is required for emergencies) at transmission hotspots. Mountainous and hilly areas, serving as primary habitats for *An minimus* and *An dirus*—the key malaria vectors in Hainan—pose great challenges. Accordingly, the CDC distributes treated or long lasting insecticidal nets and strengthens mosquito protection for people working in these areas to reduce mosquito populations and stop malaria transmission. Moreover, intermittent irrigation and other measures are implemented in irrigated rice field areas, which are habitats for *An sinensis*, a secondary vector, to reduce potential malaria transmission risks. Insecticidal vector control measures, such as insecticide treated bed nets and indoor residual spraying, were responsible for preventing 78% of clinical malaria cases in sub-Saharan Africa between 2000 and 2015.^26^ The reliance on vector control underscores the critical need for sustained investment and innovation in interventions,^27^ particularly in the face of potential lapses in drug efficacy and the ongoing threat of climate change and imported cases.

### Self-protection awareness in the population to prevent imported re-transmission

Imported cases can influence the proportion of *P falciparum* and heighten the risk of malaria transmission. As a major tourist destination, Hainan experiences a high volume of travel, which inevitably increases the risk of malaria transmission. The persistent presence of malaria vectors on the island, coupled with the frequent importation of malaria cases, heightens the risk of secondary transmission. The potential for re-establishing local transmission from imported cases remains a constant threat. Similarly, in Cape Verde, surveillance from 2010 to 2019 showed that sustained transmission of malaria could be supported only by frequent importation from outside the country. However, frequent international travel has led to a consistent influx of imported cases.^28^ These imported cases, when interacting with the local *An arabiensis* mosquito population, have resulted in secondary transmission, thereby increasing the risk of malaria resurgence in a region close to elimination.

Efforts to prevent re-transmission of imported malaria in Hainan focus on two main strategies. Firstly, health education initiatives are actively promoted to raise awareness about self-protection, particularly among people in mountainous areas where mosquito breeding is prevalent, migrant worker mobility is high, and healthcare resources are limited. This helps to encourage timely medical care and reduces the risk of local transmission from imported cases, strengthening overall disease prevention efforts. Secondly, all detected malaria cases are promptly treated within 24 hours, following standardised medication protocols by healthcare facilities at all levels. To tackle the risk of re-transmission, Hainan has participated in China’s annual inter-provincial cross examination programme since 1992. This programme aims to enhance the timeliness of diagnosis, ensure adherence to treatment protocols, strengthen collaboration between medical and preventive services, and provide up-to-date information on the risk of drug resistance.^11^

## Conclusion

The successful experience of malaria elimination in Hainan illustrates that rigorous evaluation of the effectiveness of malaria control strategies is complex but essential for informing future efforts, thereby substantially reducing the disease burden in malaria co-endemic areas. Additionally, the specific challenges faced in eliminating malaria in Hainan, such as shifts in the dominant *Plasmodium* parasites and the complexity of controlling drug resistant *Plasmodium* infections, are globally relevant, particularly in Africa, where targeting high burden areas and preventing resurgence are priorities. The lessons learnt in Hainan serve as important references for policy makers and public health practitioners in different settings.

**Key messages**

Hainan’s success in eliminating malaria lies in the long term effectiveness of a comprehensive approach
Combining indoor residual spraying with insecticide treated bed nets can ensure maximum coverage to effectively control mosquito vectors
Sustained case surveillance and continued mosquito control are needed to prevent malaria resurgence
Effective malaria elimination strategies need to be persistent but adaptable to changing transmission and intervention effectiveness, with supplementary interventions used when necessary

## Notes

Contributors and sources: HT contributed to the conceptualisation of the report. HT, CD, NCS, QL, GW, and YL were responsible for supervision of the research activity planning and execution. ZL collected and verified the data, did the statistical analyses, and wrote the manuscript. ZL and HT produced the first draft of the manuscript. HT, GW, and YL had full access to all the data in the study and discussed the results. CD and OJB provided important comments on the draft manuscript. All authors read and approved the manuscript. The corresponding author confirms that all authors have read and approved the final text.

Provenance and peer review: Commissioned; externally peer reviewed.

This article is part of a collection developed in partnership with the Chinese Center for Disease Control and Prevention, Beijing. Open access fees were funded by the Gates Foundation, China. *The BMJ* commissioned, peer reviewed, edited, and made the decision to publish. Rachael Hinton and Jocalyn Clark were the lead editors for *The BMJ*.
